# Supplementary material for: Mycoplasma Co-Infection Is Associated with Cervical Cancer Risk
Source: Cancers (Basel). 2020 Apr 28;12(5):1093. doi: 10.3390/cancers12051093 (PMC7281224; doi:10.3390/cancers12051093)
Supplement: Supplementary file 1 [file cancers-12-01093-s001.pdf]

cancers

MDPI

A

Tests of Between-Subjects Effects

Source	Dependent Variable	Type III Sum of Squares	df	Mean Square	F	Sig.	Partial Eta Squared	Noncent. Parameter	Observed Power[s]
Corrected Model	Ureaplasma spp.	1.391[a]	4	.348	1.393	.234	.006	5.573	.437
	M. hominis	.251[b]	4	.063	.279	.892	.001	1.116	.112
	M. genitalium	.052[c]	4	.013	.628	.643	.003	2.511	.207
	L. iners	1.063[d]	4	.266	1.416	.226	.006	5.665	.444
	HPV	5.389[e]	4	1.347	5.510	.000	.022	22.042	.977
	HIV	.452[f]	4	.113	.772	.543	.003	3.090	.250
	Age	456.037[g]	4	114.009	1.223	.299	.005	4.891	.386
	Last Sex	1.109[h]	4	.277	.784	.535	.003	3.137	.253
	Sex Partners	139.035[i]	4	34.759	1.947	.101	.008	7.789	.589
	Pregnancies	50.859[j]	4	12.715	2.491	.042	.010	9.963	.712
	BirthC	.979[k]	4	.245	1.113	.349	.004	4.453	.353
	Pills	1.323[l]	4	.331	1.443	.218	.006	5.773	.452
	Inject	3.834[m]	4	.959	4.038	.003	.016	16.151	.913
	Condom	.078[n]	4	.020	.498	.737	.002	1.992	.170
	Implants	.909[o]	4	.227	2.164	.071	.009	8.655	.642
	Loop	.345[p]	4	.086	1.401	.232	.006	5.603	.439
	Natural	.041[q]	4	.010	.874	.479	.003	3.497	.280
	STI	1.692[r]	4	.423	2.489	.042	.010	9.958	.712
Intercept	Ureaplasma spp.	233.363	1	233.363	934.826	.000	.484	934.826	1.000
	M. hominis	99.389	1	99.389	440.991	.000	.307	440.991	1.000
	M. genitalium	.343	1	.343	16.670	.000	.016	16.670	.983
	L. iners	462.929	1	462.929	2467.314	.000	.712	2467.314	1.000
	HPV	201.360	1	201.360	823.631	.000	.452	823.631	1.000
	HIV	26.382	1	26.382	180.247	.000	.153	180.247	1.000
	Age	1251308.347	1	1251308.347	13420.507	.000	.931	13420.507	1.000
	Last Sex	52.767	1	52.767	149.246	.000	.130	149.246	1.000
	Sex Partners	10091.962	1	10091.962	565.376	.000	.362	565.376	1.000
	Pregnancies	9836.266	1	9836.266	1926.934	.000	.659	1926.934	1.000
	BirthC	394.785	1	394.785	1794.763	.000	.643	1794.763	1.000
	Pills	109.767	1	109.767	478.975	.000	.325	478.975	1.000
	Inject	148.956	1	148.956	627.453	.000	.386	627.453	1.000
	Condom	1.459	1	1.459	37.078	.000	.036	37.078	1.000
	Implants	10.231	1	10.231	97.409	.000	.089	97.409	1.000
	Loop	4.408	1	4.408	71.687	.000	.067	71.687	1.000
	Natural	.103	1	.103	8.683	.003	.009	8.683	.837
	STI	17.795	1	17.795	104.705	.000	.095	104.705	1.000
Cytology	Ureaplasma spp.	1.391	4	.348	1.393	.234	.006	5.573	.437
	M. hominis	.251	4	.063	.279	.892	.001	1.116	.112
	M. genitalium	.052	4	.013	.628	.643	.003	2.511	.207
	L. iners	1.063	4	.266	1.416	.226	.006	5.665	.444
	HPV	5.389	4	1.347	5.510	.000	.022	22.042	.977
	HIV	.452	4	.113	.772	.543	.003	3.090	.250
	Age	456.037	4	114.009	1.223	.299	.005	4.891	.386
	Last Sex	1.109	4	.277	.784	.535	.003	3.137	.253
	Sex Partners	139.035	4	34.759	1.947	.101	.008	7.789	.589
	Pregnancies	50.859	4	12.715	2.491	.042	.010	9.963	.712
	BirthC	.979	4	.245	1.113	.349	.004	4.453	.353
	Pills	1.323	4	.331	1.443	.218	.006	5.773	.452
	Inject	3.834	4	.959	4.038	.003	.016	16.151	.913
	Condom	.078	4	.020	.498	.737	.002	1.992	.170
	Implants	.909	4	.227	2.164	.071	.009	8.655	.642
	Loop	.345	4	.086	1.401	.232	.006	5.603	.439
	Natural	.041	4	.010	.874	.479	.003	3.497	.280
	STI	1.692	4	.423	2.489	.042	.010	9.958	.712
Error	Ureaplasma spp.	248.884	997	.250					
	M. hominis	224.700	997	.225					
	M. genitalium	20.508	997	.021					
	L. iners	187.062	997	.188					
	HPV	243.745	997	.244					
	HIV	145.927	997	.146					
	Age	92958.813	997	93.239					
	Last Sex	352.496	997	.354					
	Sex Partners	17796.459	997	17.850					
	Pregnancies	5089.306	997	5.105					
	BirthC	219.305	997	.220					
	Pills	228.482	997	.229					
	Inject	236.686	997	.237					
	Condom	39.244	997	.039					
	Implants	104.720	997	.105					
	Loop	61.308	997	.061					
	Natural	11.815	997	.012					
	STI	169.441	997	.170					
Total	Ureaplasma spp.	516.000	1002						
	M. hominis	341.000	1002						
	M. genitalium	21.000	1002						
	L. iners	751.000	1002						
	HPV	464.000	1002						
	HIV	178.000	1002						
	Age	1564112.000	1002						
	Last Sex	414.000	1002						
	Sex Partners	29263.000	1002						
	Pregnancies	16187.000	1002						
	BirthC	675.000	1002						
	Pills	357.000	1002						
	Inject	401.000	1002						
	Condom	41.000	1002						
	Implants	120.000	1002						
	Loop	66.000	1002						
	Natural	12.000	1002						
	STI	188.000	1002						
Corrected Total	Ureaplasma spp.	250.275	1001						
	M. hominis	224.951	1001						
	M. genitalium	20.560	1001						
	L. iners	188.125	1001						
	HPV	249.134	1001						
	HIV	146.379	1001						
	Age	93414.850	1001						
	Last Sex	353.605	1001						
	Sex Partners	17935.494	1001						
	Pregnancies	5140.165	1001						
	BirthC	220.284	1001						
	Pills	229.805	1001						
	Inject	240.520	1001						
	Condom	39.322	1001						
	Implants	105.629	1001						
	Loop	61.653	1001						
	Natural	11.856	1001						
	STI	171.134	1001						

a. R Squared = .006 (Adjusted R Squared = .002)
b. R Squared = .001 (Adjusted R Squared = -.003)
c. R Squared = .003 (Adjusted R Squared = -.001)
d. R Squared = .006 (Adjusted R Squared = .002)
e. R Squared = .022 (Adjusted R Squared = .018)
f. R Squared = .003 (Adjusted R Squared = -.001)
g. R Squared = .005 (Adjusted R Squared = .001)
h. R Squared = .003 (Adjusted R Squared = -.001)
i. R Squared = .008 (Adjusted R Squared = .004)
j. R Squared = .010 (Adjusted R Squared = .006)
k. R Squared = .004 (Adjusted R Squared = .000)
l. R Squared = .006 (Adjusted R Squared = .002)
m. R Squared = .016 (Adjusted R Squared = .012)
n. R Squared = .002 (Adjusted R Squared = -.002)
o. R Squared = .009 (Adjusted R Squared = .005)
p. R Squared = .006 (Adjusted R Squared = .002)
q. R Squared = .003 (Adjusted R Squared = -.001)
r. R Squared = .010 (Adjusted R Squared = .006)
s. Computed using alpha = .05

B

Tests of Between-Subjects Effects

Source	Dependent Variable	Type III Sum of Squares	df	Mean Square	F	Sig.	Partial Eta Squared	Noncent. Parameter	Observed Power[s]
Corrected Model	Cytology	2.639[a]	1	2.639	1.775	.183	.002	1.775	.265
	M. hominis	2.004[b]	1	2.004	8.989	.003	.009	8.989	.850
	M. genitalium	.041[c]	1	.041	1.976	.160	.002	1.976	.290
	L. iners	3.190[d]	1	3.190	17.252	.000	.017	17.252	.986
	HPV	.328[e]	1	.328	1.316	.252	.001	1.316	.209
	HIV	1.066[f]	1	1.066	7.337	.007	.007	7.337	.772
	Age	38.116[g]	1	38.116	.408	.523	.000	.408	.098
	Last Sex	1.112[h]	1	1.112	3.155	.076	.003	3.155	.427
	Sex Partners	.147[i]	1	.147	.008	.928	.000	.008	.051
	Pregnancies	.130[j]	1	.130	.025	.874	.000	.025	.053
	BirthC	.077[k]	1	.077	.351	.554	.000	.351	.091
	Pills	.003[l]	1	.003	.012	.911	.000	.012	.051
	Inject	.528[m]	1	.528	2.201	.138	.002	2.201	.317
	Condom	.068[n]	1	.068	1.723	.190	.002	1.723	.259
	Implants	.207[o]	1	.207	1.967	.161	.002	1.967	.289
	Loop	.064[p]	1	.064	1.032	.310	.001	1.032	.174
	Natural	.006[q]	1	.006	.469	.494	.000	.469	.105
	STI	.062[r]	1	.062	.364	.547	.000	.364	.093
Intercept	Cytology	2842.655	1	2842.655	1911.721	.000	.657	1911.721	1.000
	M. hominis	115.034	1	115.034	515.969	.000	.340	515.969	1.000
	M. genitalium	.432	1	.432	21.042	.000	.021	21.042	.996
	L. iners	559.837	1	559.837	3027.221	.000	.752	3027.221	1.000
	HPV	214.172	1	214.172	860.798	.000	.463	860.798	1.000
	HIV	31.246	1	31.246	215.024	.000	.177	215.024	1.000
	Age	1469826.967	1	1469826.967	15740.826	.000	.940	15740.826	1.000
	Last Sex	60.833	1	60.833	172.578	.000	.147	172.578	1.000
	Sex Partners	11314.909	1	11314.909	630.872	.000	.387	630.872	1.000
	Pregnancies	11034.668	1	11034.668	2146.808	.000	.682	2146.808	1.000
	BirthC	453.953	1	453.953	2061.483	.000	.673	2061.483	1.000
	Pills	127.117	1	127.117	553.156	.000	.356	553.156	1.000
	Inject	159.786	1	159.786	665.796	.000	.400	665.796	1.000
	Condom	1.696	1	1.696	43.214	.000	.041	43.214	1.000
	Implants	14.255	1	14.255	135.222	.000	.119	135.222	1.000
	Loop	4.375	1	4.375	71.034	.000	.066	71.034	1.000
	Natural	.145	1	.145	12.259	.000	.012	12.259	.938
	STI	16.913	1	16.913	98.862	.000	.090	98.862	1.000
Ureaplasmaspp	Cytology	2.639	1	2.639	1.775	.183	.002	1.775	.265
	M. hominis	2.004	1	2.004	8.989	.003	.009	8.989	.850
	M. genitalium	.041	1	.041	1.976	.160	.002	1.976	.290
	L. iners	3.190	1	3.190	17.252	.000	.017	17.252	.986
	HPV	.328	1	.328	1.316	.252	.001	1.316	.209
	HIV	1.066	1	1.066	7.337	.007	.007	7.337	.772
	Age	38.116	1	38.116	.408	.523	.000	.408	.098
	Last Sex	1.112	1	1.112	3.155	.076	.003	3.155	.427
	Sex Partners	.147	1	.147	.008	.928	.000	.008	.051
	Pregnancies	.130	1	.130	.025	.874	.000	.025	.053
	BirthC	.077	1	.077	.351	.554	.000	.351	.091
	Pills	.003	1	.003	.012	.911	.000	.012	.051
	Inject	.528	1	.528	2.201	.138	.002	2.201	.317
	Condom	.068	1	.068	1.723	.190	.002	1.723	.259
	Implants	.207	1	.207	1.967	.161	.002	1.967	.289
	Loop	.064	1	.064	1.032	.310	.001	1.032	.174
	Natural	.006	1	.006	.469	.494	.000	.469	.105
	STI	.062	1	.062	.364	.547	.000	.364	.093
Error	Cytology	1486.962	1000	1.487					
	M. hominis	222.947	1000	.223					
	M. genitalium	20.519	1000	.021					
	L. iners	184.934	1000	.185					
	HPV	248.806	1000	.249					
	HIV	145.313	1000	.145					
	Age	93376.734	1000	93.377					
	Last Sex	352.493	1000	.352					
	Sex Partners	17935.347	1000	17.935					
	Pregnancies	5140.035	1000	5.140					
	BirthC	220.207	1000	.220					
	Pills	229.803	1000	.230					
	Inject	239.992	1000	.240					
	Condom	39.255	1000	.039					
	Implants	105.421	1000	.105					
	Loop	61.589	1000	.062					
	Natural	11.851	1000	.012					
	STI	171.072	1000	.171					
Total	Cytology	4340.000	1002						
	M. hominis	341.000	1002						
	M. genitalium	21.000	1002						
	L. iners	751.000	1002						
	HPV	464.000	1002						
	HIV	178.000	1002						
	Age	1564112.000	1002						
	Last Sex	414.000	1002						
	Sex Partners	29263.000	1002						
	Pregnancies	16187.000	1002						
	BirthC	675.000	1002						
	Pills	357.000	1002						
	Inject	401.000	1002						
	Condom	41.000	1002						
	Implants	120.000	1002						
	Loop	66.000	1002						
	Natural	12.000	1002						
	STI	188.000	1002						
Corrected Total	Cytology	1489.601	1001						
	M. hominis	224.951	1001						
	M. genitalium	20.560	1001						
	L. iners	188.125	1001						
	HPV	249.134	1001						
	HIV	146.379	1001						
	Age	93414.850	1001						
	Last Sex	353.605	1001						
	Sex Partners	17935.494	1001						
	Pregnancies	5140.165	1001						
	BirthC	220.284	1001						
	Pills	229.805	1001						
	Inject	240.520	1001						
	Condom	39.322	1001						
	Implants	105.629	1001						
	Loop	61.653	1001						
	Natural	11.856	1001						
	STI	171.134	1001						

a. R Squared = .002 (Adjusted R Squared = .001)
b. R Squared = .009 (Adjusted R Squared = .008)
c. R Squared = .002 (Adjusted R Squared = .001)
d. R Squared = .017 (Adjusted R Squared = .016)
e. R Squared = .001 (Adjusted R Squared = .000)
f. R Squared = .007 (Adjusted R Squared = .006)
g. R Squared = .000 (Adjusted R Squared = -.001)
h. R Squared = .003 (Adjusted R Squared = .002)
i. R Squared = .000 (Adjusted R Squared = -.001)
j. R Squared = .000 (Adjusted R Squared = -.001)
k. R Squared = .000 (Adjusted R Squared = -.001)
l. R Squared = .000 (Adjusted R Squared = -.001)
m. R Squared = .002 (Adjusted R Squared = .001)
n. R Squared = .002 (Adjusted R Squared = .001)
o. R Squared = .002 (Adjusted R Squared = .001)
p. R Squared = .001 (Adjusted R Squared = .000)
q. R Squared = .000 (Adjusted R Squared = -.001)
r. R Squared = .000 (Adjusted R Squared = -.001)
s. Computed using alpha = .05

2

C

Tests of Between-Subjects Effects

Source	Dependent Variable	Type III Sum of Squares	df	Mean Square	F	Sig.	Partial Eta Squared	Noncent. Parameter	Observed Power[s]
Corrected Model	Cytology	.036[a]	1	.036	.024	.876	.000	.024	.053
	Ureaplasma spp.	2.230[b]	1	2.230	8.989	.003	.009	8.989	.850
	M. genitalium	.105[c]	1	.105	5.119	.024	.005	5.119	.618
	L. iners	3.848[d]	1	3.848	20.880	.000	.020	20.880	.995
	HPV	6.450[e]	1	6.450	26.579	.000	.026	26.579	.999
	HIV	13.167[f]	1	13.167	98.840	.000	.090	98.840	1.000
	Age	165.895[g]	1	165.895	1.779	.183	.002	1.779	.266
	Last Sex	1.396[h]	1	1.396	3.962	.047	.004	3.962	.511
	Sex Partners	99.308[i]	1	99.308	5.568	.018	.006	5.568	.654
	Pregnancies	5.212[j]	1	5.212	1.015	.314	.001	1.015	.172
	BirthC	.002[k]	1	.002	.010	.919	.000	.010	.051
	Pills	.028[l]	1	.028	.121	.727	.000	.121	.064
	Inject	.136[m]	1	.136	.566	.452	.001	.566	.117
	Condom	.019[n]	1	.019	.474	.491	.000	.474	.106
	Implants	.021[o]	1	.021	.197	.657	.000	.197	.073
	Loop	.133[p]	1	.133	2.155	.142	.002	2.155	.311
	Natural	3.124E-5[q]	1	3.124E-5	.003	.959	.000	.003	.050
	STI	.841[r]	1	.841	4.941	.026	.005	4.941	.603
Intercept	Cytology	2565.849	1	2565.849	1722.550	.000	.633	1722.550	1.000
	Ureaplasma spp.	253.583	1	253.583	1022.322	.000	.506	1022.322	1.000
	M. genitalium	.536	1	.536	26.196	.000	.026	26.196	.999
	L. iners	534.027	1	534.027	2897.959	.000	.743	2897.959	1.000
	HPV	216.143	1	216.143	890.637	.000	.471	890.637	1.000
	HIV	42.089	1	42.089	315.953	.000	.240	315.953	1.000
	Age	1311260.933	1	1311260.933	14061.937	.000	.934	14061.937	1.000
	Last Sex	48.821	1	48.821	138.613	.000	.122	138.613	1.000
	Sex Partners	10824.290	1	10824.290	606.872	.000	.378	606.872	1.000
	Pregnancies	9775.440	1	9775.440	1903.706	.000	.656	1903.706	1.000
	BirthC	407.723	1	407.723	1850.912	.000	.649	1850.912	1.000
	Pills	115.365	1	115.365	502.074	.000	.334	502.074	1.000
	Inject	146.954	1	146.954	611.332	.000	.379	611.332	1.000
	Condom	1.615	1	1.615	41.101	.000	.039	41.101	1.000
	Implants	13.238	1	13.238	125.354	.000	.111	125.354	1.000
	Loop	3.458	1	3.458	56.208	.000	.053	56.208	1.000
	Natural	.128	1	.128	10.777	.001	.011	10.777	.907
	STI	17.512	1	17.512	102.836	.000	.093	102.836	1.000
M.hominis	Cytology	.036	1	.036	.024	.876	.000	.024	.053
	Ureaplasma spp.	2.230	1	2.230	8.989	.003	.009	8.989	.850
	M. genitalium	.105	1	.105	5.119	.024	.005	5.119	.618
	L. iners	3.848	1	3.848	20.880	.000	.020	20.880	.995
	HPV	6.450	1	6.450	26.579	.000	.026	26.579	.999
	HIV	13.167	1	13.167	98.840	.000	.090	98.840	1.000
	Age	165.895	1	165.895	1.779	.183	.002	1.779	.266
	Last Sex	1.396	1	1.396	3.962	.047	.004	3.962	.511
	Sex Partners	99.308	1	99.308	5.568	.018	.006	5.568	.654
	Pregnancies	5.212	1	5.212	1.015	.314	.001	1.015	.172
	BirthC	.002	1	.002	.010	.919	.000	.010	.051
	Pills	.028	1	.028	.121	.727	.000	.121	.064
	Inject	.136	1	.136	.566	.452	.001	.566	.117
	Condom	.019	1	.019	.474	.491	.000	.474	.106
	Implants	.021	1	.021	.197	.657	.000	.197	.073
	Loop	.133	1	.133	2.155	.142	.002	2.155	.311
	Natural	3.124E-5	1	3.124E-5	.003	.959	.000	.003	.050
	STI	.841	1	.841	4.941	.026	.005	4.941	.603
Error	Cytology	1489.564	1000	1.490					
	Ureaplasma spp.	248.046	1000	.248					
	M. genitalium	20.455	1000	.020					
	L. iners	184.277	1000	.184					
	HPV	242.684	1000	.243					
	HIV	133.212	1000	.133					
	Age	93248.955	1000	93.249					
	Last Sex	352.209	1000	.352					
	Sex Partners	17836.186	1000	17.836					
	Pregnancies	5134.952	1000	5.135					
	BirthC	220.282	1000	.220					
	Pills	229.777	1000	.230					
	Inject	240.384	1000	.240					
	Condom	39.304	1000	.039					
	Implants	105.608	1000	.106					
	Loop	61.520	1000	.062					
	Natural	11.856	1000	.012					
	STI	170.292	1000	.170					
Total	Cytology	4340.000	1002						
	Ureaplasma spp.	516.000	1002						
	M. genitalium	21.000	1002						
	L. iners	751.000	1002						
	HPV	464.000	1002						
	HIV	178.000	1002						
	Age	1564112.000	1002						
	Last Sex	414.000	1002						
	Sex Partners	29263.000	1002						
	Pregnancies	16187.000	1002						
	BirthC	675.000	1002						
	Pills	357.000	1002						
	Inject	401.000	1002						
	Condom	41.000	1002						
	Implants	120.000	1002						
	Loop	66.000	1002						
	Natural	12.000	1002						
	STI	188.000	1002						
Corrected Total	Cytology	1489.601	1001						
	Ureaplasma spp.	250.275	1001						
	M. genitalium	20.560	1001						
	L. iners	188.125	1001						
	HPV	249.134	1001						
	HIV	146.379	1001						
	Age	93414.850	1001						
	Last Sex	353.605	1001						
	Sex Partners	17935.494	1001						
	Pregnancies	5140.165	1001						
	BirthC	220.284	1001						
	Pills	229.805	1001						
	Inject	240.520	1001						
	Condom	39.322	1001						
	Implants	105.629	1001						
	Loop	61.653	1001						
	Natural	11.856	1001						
	STI	171.134	1001						

a. R Squared = .000 (Adjusted R Squared = -.001)
b. R Squared = .009 (Adjusted R Squared = .008)
c. R Squared = .005 (Adjusted R Squared = .004)
d. R Squared = .020 (Adjusted R Squared = .019)
e. R Squared = .026 (Adjusted R Squared = .025)
f. R Squared = .090 (Adjusted R Squared = .089)
g. R Squared = .002 (Adjusted R Squared = .001)
h. R Squared = .004 (Adjusted R Squared = .003)
i. R Squared = .006 (Adjusted R Squared = .005)
j. R Squared = .001 (Adjusted R Squared = .000)
k. R Squared = .000 (Adjusted R Squared = -.001)
l. R Squared = .000 (Adjusted R Squared = -.001)
m. R Squared = .001 (Adjusted R Squared = .000)
n. R Squared = .000 (Adjusted R Squared = -.001)
o. R Squared = .000 (Adjusted R Squared = -.001)
p. R Squared = .002 (Adjusted R Squared = .001)
q. R Squared = .000 (Adjusted R Squared = -.001)
r. R Squared = .005 (Adjusted R Squared = .004)
s. Computed using alpha = .05

3

D

Tests of Between-Subjects Effects

Source	Dependent Variable	Type III Sum of Squares	df	Mean Square	F	Sig.	Partial Eta Squared	Noncent. Parameter	Observed Power[s]
Corrected Model	Cytology	.009[a]	1	.009	.006	.940	.000	.006	.051
	Ureaplasma spp.	.494[b]	1	.494	1.976	.160	.002	1.976	.290
	M. hominis	1.146[c]	1	1.146	5.119	.024	.005	5.119	.618
	L. iners	.077[d]	1	.077	.411	.522	.000	.411	.098
	HPV	.026[e]	1	.026	.103	.749	.000	.103	.062
	HIV	1.912[f]	1	1.912	13.233	.000	.013	13.233	.953
	Age	178.258[g]	1	178.258	1.912	.167	.002	1.912	.282
	Last Sex	.226[h]	1	.226	.640	.424	.001	.640	.126
	Sex Partners	.007[i]	1	.007	.000	.984	.000	.000	.050
	Pregnancies	1.087[j]	1	1.087	.212	.646	.000	.212	.075
	BirthC	.064[k]	1	.064	.290	.590	.000	.290	.084
	Pills	.300[l]	1	.300	1.306	.253	.001	1.306	.208
	Inject	.124[m]	1	.124	.515	.473	.001	.515	.111
	Condom	.063[n]	1	.063	1.612	.204	.002	1.612	.245
	Implants	.013[o]	1	.013	.122	.727	.000	.122	.064
	Loop	.019[p]	1	.019	.300	.584	.000	.300	.085
	Natural	.003[q]	1	.003	.260	.611	.000	.260	.080
	STI	1.915[r]	1	1.915	11.319	.001	.011	11.319	.919
Intercept	Cytology	231.246	1	231.246	155.241	.000	.134	155.241	1.000
	Ureaplasma spp.	28.549	1	28.549	114.298	.000	.103	114.298	1.000
	M. hominis	16.906	1	16.906	75.539	.000	.070	75.539	1.000
	L. iners	49.890	1	49.890	265.303	.000	.210	265.303	1.000
	HPV	16.373	1	16.373	65.726	.000	.062	65.726	1.000
	HIV	8.618	1	8.618	59.656	.000	.056	59.656	1.000
	Age	111983.192	1	111983.192	1201.065	.000	.546	1201.065	1.000
	Last Sex	3.136	1	3.136	8.875	.003	.009	8.875	.845
	Sex Partners	934.770	1	934.770	52.118	.000	.050	52.118	1.000
	Pregnancies	847.514	1	847.514	164.916	.000	.142	164.916	1.000
	BirthC	34.419	1	34.419	156.294	.000	.135	156.294	1.000
	Pills	7.326	1	7.326	31.919	.000	.031	31.919	1.000
	Inject	15.733	1	15.733	65.445	.000	.061	65.445	1.000
	Condom	.375	1	.375	9.543	.002	.009	9.543	.870
	Implants	.955	1	.955	9.042	.003	.009	9.042	.852
	Loop	.529	1	.529	8.591	.003	.009	8.591	.834
	Natural	.003	1	.003	.260	.611	.000	.260	.080
	STI	6.263	1	6.263	37.010	.000	.036	37.010	1.000
M.genitalium	Cytology	.009	1	.009	.006	.940	.000	.006	.051
	Ureaplasma spp.	.494	1	.494	1.976	.160	.002	1.976	.290
	M. hominis	1.146	1	1.146	5.119	.024	.005	5.119	.618
	L. iners	.077	1	.077	.411	.522	.000	.411	.098
	HPV	.026	1	.026	.103	.749	.000	.103	.062
	HIV	1.912	1	1.912	13.233	.000	.013	13.233	.953
	Age	178.258	1	178.258	1.912	.167	.002	1.912	.282
	Last Sex	.226	1	.226	.640	.424	.001	.640	.126
	Sex Partners	.007	1	.007	.000	.984	.000	.000	.050
	Pregnancies	1.087	1	1.087	.212	.646	.000	.212	.075
	BirthC	.064	1	.064	.290	.590	.000	.290	.084
	Pills	.300	1	.300	1.306	.253	.001	1.306	.208
	Inject	.124	1	.124	.515	.473	.001	.515	.111
	Condom	.063	1	.063	1.612	.204	.002	1.612	.245
	Implants	.013	1	.013	.122	.727	.000	.122	.064
	Loop	.019	1	.019	.300	.584	.000	.300	.085
	Natural	.003	1	.003	.260	.611	.000	.260	.080
	STI	1.915	1	1.915	11.319	.001	.011	11.319	.919
Error	Cytology	1489.592	1000	1.490					
	Ureaplasma spp.	249.782	1000	.250					
	M. hominis	223.805	1000	.224					
	L. iners	188.047	1000	.188					
	HPV	249.108	1000	.249					
	HIV	144.467	1000	.144					
	Age	93236.592	1000	93.237					
	Last Sex	353.379	1000	.353					
	Sex Partners	17935.487	1000	17.935					
	Pregnancies	5139.078	1000	5.139					
	BirthC	220.220	1000	.220					
	Pills	229.506	1000	.230					
	Inject	240.396	1000	.240					
	Condom	39.259	1000	.039					
	Implants	105.616	1000	.106					
	Loop	61.634	1000	.062					
	Natural	11.853	1000	.012					
	STI	169.218	1000	.169					
Total	Cytology	4340.000	1002						
	Ureaplasma spp.	516.000	1002						
	M. hominis	341.000	1002						
	L. iners	751.000	1002						
	HPV	464.000	1002						
	HIV	178.000	1002						
	Age	1564112.000	1002						
	Last Sex	414.000	1002						
	Sex Partners	29263.000	1002						
	Pregnancies	16187.000	1002						
	BirthC	675.000	1002						
	Pills	357.000	1002						
	Inject	401.000	1002						
	Condom	41.000	1002						
	Implants	120.000	1002						
	Loop	66.000	1002						
	Natural	12.000	1002						
	STI	188.000	1002						
Corrected Total	Cytology	1489.601	1001						
	Ureaplasma spp.	250.275	1001						
	M. hominis	224.951	1001						
	L. iners	188.125	1001						
	HPV	249.134	1001						
	HIV	146.379	1001						
	Age	93414.850	1001						
	Last Sex	353.605	1001						
	Sex Partners	17935.494	1001						
	Pregnancies	5140.165	1001						
	BirthC	220.284	1001						
	Pills	229.805	1001						
	Inject	240.520	1001						
	Condom	39.322	1001						
	Implants	105.629	1001						
	Loop	61.653	1001						
	Natural	11.856	1001						
	STI	171.134	1001						

a. R Squared = .000 (Adjusted R Squared = -.001)
b. R Squared = .002 (Adjusted R Squared = .001)
c. R Squared = .005 (Adjusted R Squared = .004)
d. R Squared = .000 (Adjusted R Squared = -.001)
e. R Squared = .000 (Adjusted R Squared = -.001)
f. R Squared = .013 (Adjusted R Squared = .012)
g. R Squared = .002 (Adjusted R Squared = .001)
h. R Squared = .001 (Adjusted R Squared = .000)
i. R Squared = .000 (Adjusted R Squared = -.001)
j. R Squared = .000 (Adjusted R Squared = -.001)
k. R Squared = .000 (Adjusted R Squared = -.001)
l. R Squared = .001 (Adjusted R Squared = .000)
m. R Squared = .001 (Adjusted R Squared = .000)
n. R Squared = .002 (Adjusted R Squared = .001)
o. R Squared = .000 (Adjusted R Squared = -.001)
p. R Squared = .000 (Adjusted R Squared = -.001)
q. R Squared = .000 (Adjusted R Squared = -.001)
r. R Squared = .011 (Adjusted R Squared = .010)
s. Computed using alpha = .05

4

E

Tests of Between-Subjects Effects

Source	Dependent Variable	Type III Sum of Squares	df	Mean Square	F	Sig.	Partial Eta Squared	Noncent. Parameter	Observed Power[s]
Corrected Model	Cytology	2.975[a]	1	2.975	2.001	.158	.002	2.001	.293
	M. genitalium	.008[b]	1	.008	.411	.522	.000	.411	.098
	Ureaplasma spp.	4.244[c]	1	4.244	17.252	.000	.017	17.252	.986
	M. hominis	4.601[d]	1	4.601	20.880	.000	.020	20.880	.995
	HPV	5.185[e]	1	5.185	21.254	.000	.021	21.254	.996
	HIV	.714[f]	1	.714	4.901	.027	.005	4.901	.599
	Age	2314.393[g]	1	2314.393	25.405	.000	.025	25.405	.999
	Last Sex	3.423[h]	1	3.423	9.776	.002	.010	9.776	.878
	Sex Partners	38.343[i]	1	38.343	2.142	.144	.002	2.142	.310
	Pregnancies	54.860[j]	1	54.860	10.788	.001	.011	10.788	.907
	BirthC	.348[k]	1	.348	1.581	.209	.002	1.581	.241
	Pills	.001[l]	1	.001	.004	.948	.000	.004	.050
	Inject	.580[m]	1	.580	2.419	.120	.002	2.419	.343
	Condom	.057[n]	1	.057	1.448	.229	.001	1.448	.225
	Implants	.050[o]	1	.050	.471	.493	.000	.471	.105
	Loop	.106[p]	1	.106	1.723	.190	.002	1.723	.259
	Natural	.021[q]	1	.021	1.807	.179	.002	1.807	.269
	STI	.011[r]	1	.011	.064	.800	.000	.064	.057
Intercept	Cytology	2221.023	1	2221.023	1494.002	.000	.599	1494.002	1.000
	M. genitalium	.280	1	.280	13.620	.000	.013	13.620	.958
	Ureaplasma spp.	171.570	1	171.570	697.350	.000	.411	697.350	1.000
	M. hominis	68.313	1	68.313	310.022	.000	.237	310.022	1.000
	HPV	133.788	1	133.788	548.425	.000	.354	548.425	1.000
	HIV	19.816	1	19.816	136.036	.000	.120	136.036	1.000
	Age	1155523.622	1	1155523.622	12684.059	.000	.927	12684.059	1.000
	Last Sex	58.645	1	58.645	167.470	.000	.143	167.470	1.000
	Sex Partners	7946.487	1	7946.487	444.008	.000	.307	444.008	1.000
	Pregnancies	8983.083	1	8983.083	1766.479	.000	.639	1766.479	1.000
	BirthC	330.703	1	330.703	1503.627	.000	.601	1503.627	1.000
	Pills	95.219	1	95.219	414.346	.000	.293	414.346	1.000
	Inject	112.317	1	112.317	468.106	.000	.319	468.106	1.000
	Condom	1.007	1	1.007	25.645	.000	.025	25.645	.999
	Implants	10.074	1	10.074	95.414	.000	.087	95.414	1.000
	Loop	3.879	1	3.879	63.018	.000	.059	63.018	1.000
	Natural	.065	1	.065	5.518	.019	.005	5.518	.651
	STI	13.041	1	13.041	76.208	.000	.071	76.208	1.000
L.iners	Cytology	2.975	1	2.975	2.001	.158	.002	2.001	.293
	M. genitalium	.008	1	.008	.411	.522	.000	.411	.098
	Ureaplasma spp.	4.244	1	4.244	17.252	.000	.017	17.252	.986
	M. hominis	4.601	1	4.601	20.880	.000	.020	20.880	.995
	HPV	5.185	1	5.185	21.254	.000	.021	21.254	.996
	HIV	.714	1	.714	4.901	.027	.005	4.901	.599
	Age	2314.393	1	2314.393	25.405	.000	.025	25.405	.999
	Last Sex	3.423	1	3.423	9.776	.002	.010	9.776	.878
	Sex Partners	38.343	1	38.343	2.142	.144	.002	2.142	.310
	Pregnancies	54.860	1	54.860	10.788	.001	.011	10.788	.907
	BirthC	.348	1	.348	1.581	.209	.002	1.581	.241
	Pills	.001	1	.001	.004	.948	.000	.004	.050
	Inject	.580	1	.580	2.419	.120	.002	2.419	.343
	Condom	.057	1	.057	1.448	.229	.001	1.448	.225
	Implants	.050	1	.050	.471	.493	.000	.471	.105
	Loop	.106	1	.106	1.723	.190	.002	1.723	.259
	Natural	.021	1	.021	1.807	.179	.002	1.807	.269
	STI	.011	1	.011	.064	.800	.000	.064	.057
Error	Cytology	1486.626	1000	1.487					
	M. genitalium	20.551	1000	.021					
	Ureaplasma spp.	246.031	1000	.246					
	M. hominis	220.350	1000	.220					
	HPV	243.949	1000	.244					
	HIV	145.665	1000	.146					
	Age	91100.458	1000	91.100					
	Last Sex	350.182	1000	.350					
	Sex Partners	17897.151	1000	17.897					
	Pregnancies	5085.305	1000	5.085					
	BirthC	219.937	1000	.220					
	Pills	229.804	1000	.230					
	Inject	239.939	1000	.240					
	Condom	39.265	1000	.039					
	Implants	105.579	1000	.106					
	Loop	61.547	1000	.062					
	Natural	11.835	1000	.012					
	STI	171.123	1000	.171					
Total	Cytology	4340.000	1002						
	M. genitalium	21.000	1002						
	Ureaplasma spp.	516.000	1002						
	M. hominis	341.000	1002						
	HPV	464.000	1002						
	HIV	178.000	1002						
	Age	1564112.000	1002						
	Last Sex	414.000	1002						
	Sex Partners	29263.000	1002						
	Pregnancies	16187.000	1002						
	BirthC	675.000	1002						
	Pills	357.000	1002						
	Inject	401.000	1002						
	Condom	41.000	1002						
	Implants	120.000	1002						
	Loop	66.000	1002						
	Natural	12.000	1002						
	STI	188.000	1002						
Corrected Total	Cytology	1489.601	1001						
	M. genitalium	20.560	1001						
	Ureaplasma spp.	250.275	1001						
	M. hominis	224.951	1001						
	HPV	249.134	1001						
	HIV	146.379	1001						
	Age	93414.850	1001						
	Last Sex	353.605	1001						
	Sex Partners	17935.494	1001						
	Pregnancies	5140.165	1001						
	BirthC	220.284	1001						
	Pills	229.805	1001						
	Inject	240.520	1001						
	Condom	39.322	1001						
	Implants	105.629	1001						
	Loop	61.653	1001						
	Natural	11.856	1001						
	STI	171.134	1001						

a. R Squared = .002 (Adjusted R Squared = .001)
b. R Squared = .000 (Adjusted R Squared = -.001)
c. R Squared = .017 (Adjusted R Squared = .016)
d. R Squared = .020 (Adjusted R Squared = .019)
e. R Squared = .021 (Adjusted R Squared = .020)
f. R Squared = .005 (Adjusted R Squared = .004)
g. R Squared = .025 (Adjusted R Squared = .024)
h. R Squared = .010 (Adjusted R Squared = .009)
i. R Squared = .002 (Adjusted R Squared = .001)
j. R Squared = .011 (Adjusted R Squared = .010)
k. R Squared = .002 (Adjusted R Squared = .001)
l. R Squared = .000 (Adjusted R Squared = -.001)
m. R Squared = .002 (Adjusted R Squared = .001)
n. R Squared = .001 (Adjusted R Squared = .000)
o. R Squared = .000 (Adjusted R Squared = -.001)
p. R Squared = .002 (Adjusted R Squared = .001)
q. R Squared = .002 (Adjusted R Squared = .001)
r. R Squared = .000 (Adjusted R Squared = -.001)
s. Computed using alpha = .05

Figure S1. MANOVA Results. (**A**). MANOVA analysis of factors in relationship cervical cytology; (**B**). MANOVA analysis of factors in relationship to *Ureaplasma spp.* detection; (**C**). MANOVA analysis of factors in relationship to *M. hominis* detection; (**D**). MANOVA analysis of factors in relationship to *M. genitalium* detection; (**E**). MANOVA analysis of factors in relationship to *L. iners* detection.

© 2020 by the authors. Submitted for possible open access publication under the terms and conditions of the Creative Commons Attribution (CC BY) license (http://creativecommons.org/licenses/by/4.0/).
